# Shear Wave Elastography Evaluation of Testicular Stiffness in Dogs Affected by Testicular Pathology

**DOI:** 10.3390/ani15030353

**Published:** 2025-01-26

**Authors:** Tiziana Caspanello, Viola Zappone, Riccardo Orlandi, Monica Sforna, Cristano Boiti, Letizia Sinagra, Giulia Donato, Massimo De Majo, Nicola Maria Iannelli, Alessandro Troisi

**Affiliations:** 1 Department of Veterinary Sciences, University of Messina, Viale Palatucci 23, 98168 Messina, Italy; viola.zappone@unime.it (V.Z.); mdemajo@unime.it (M.D.M.); nicola_iannelli@libero.it (N.M.I.); 2 Clinica Veterinaria Anicura Tyrus, Via Bartocci 1G, 05100 Terni, Italy; 3 Department of Veterinary Medicine, University of Perugia, Via San Costanzo 4, 06126 Perugia, Italy; monica.sforna@unipg.it; 4 Tyrus Science Foundation, Via Bartocci 1G, 05100 Terni, Italy; boiti.cristiano@gmail.com; 5 Independent Researcher, 95030 Mascalucia, Italy; letizia.sinagra@gmail.com; 6 Independent Researcher, 88040 Martelletto, Italy; giudonato@unime.it; 7 Clinica Veterinaria Camagna—VetPartners, Via Fortunato Licandro 13, 89124 Reggio di Calabria, Italy; 8 School of Biosciences and Veterinary Medicine, University of Camerino, Via Circonvallazione 93/95, 62024 Macerata, Italy; alessandro.troisi@unicam.it

**Keywords:** shear wave elastography, ultrasound, dog, testis, testicular pathologies

## Abstract

Shear wave elastography (SWE) is an advanced ultrasound technique that assesses tissue stiffness by measuring shear wave speed (SWS) produced after an acoustic impulse. It includes bidimensional (2D-SWE) and focal point (pSWE) methods, allowing qualitative and quantitative analysis of tissue stiffness. This study aimed to describe the elastographic features of testicular abnormalities in dogs, supported by histological findings. Eighteen dogs with testicular abnormalities underwent B-mode ultrasound, power and color Doppler ultrasound, 2D-SWE, and pSWE before orchiectomy. Five cryptorchid testes were excluded and thirty-one testes (12 normal, 7 with leydigomas, 6 with seminomas, 1 with a round cell tumor, and 5 with orchitis) were examined. Normal testes, lesions, and adjacent healthy tissues (no evident ultrasound changes, NEUC) were sampled. Testicular abnormalities presented SWS values of 1.05–4.89 m/s (2D-SWE) and 1.35–5.31 m/s (pSWE). Significant differences were observed among normal testes, NEUC areas, and those with orchitis, leydigomas, and seminomas by both 2D-SWE and pSWE. Normal testes were significantly softer than ones with leydigomas, seminomas, and orchitis, and NEUC areas also had different SWS values compared to those with tumors and orchitis (*p* < 0.05). However, SWE techniques lacked specificity in differentiating between orchitis and tumors. Diagnostic accuracy of SWE techniques for testicular lesions remains challenging and requires further investigation to fully address its clinical potential.

## Simple Summary

The aim of this study was to evaluate the use of 2D-SWE and pSWE for the diagnosis of testicular abnormalities in dogs, to describe their elastographic features, and to assess the diagnostic potential of ultrasound techniques with respect to the histological diagnosis of diseased canine testes. Eighteen male dogs, presented to a veterinary clinic for surgical orchiectomy, were included in the study. Prior to surgical excision, the testes were examined using B-mode US, color Doppler US, and 2D-SWE and pSWE techniques. The results of our study revealed significant differences in SWS values between healthy and pathological tissues. Although further studies are needed to clarify the role of SWE in the characterization of testicular lesions in dogs, the results suggest that the method may become a useful diagnostic tool.

## 1. Introduction

The assessment of testicular disease in veterinary medicine presents significant challenges in clinical practice due to the variety of pathologies that can affect this organ [1]. Testicular ultrasound is an important and complementary diagnostic technique for the detection of reproductive disorders in dogs [2,3], since it provides anatomical details of the testes and surrounding structures [4] and enables the detection of lesions that are too small to be detected by palpation [5]. However, the specificity of conventional ultrasound alone remains rather poor [6]. Therefore, new ultrasound techniques, such as Doppler, contrast-enhanced ultrasound (CEUS), 3D/4D ultrasound, and elastography, have been developed to improve the diagnostic power of ultrasound in several diagnostic settings of the veterinary field. In fact, to date, thanks to the combined use of traditional and modern ultrasound techniques, the multiparametric evaluation represents the most accurate diagnostic strategy for assessing reproductive health both in dogs and humans [3,6].

Ultrasound elastography is an advanced ultrasound technique that enables the measurement of tissue stiffness. It provides information on the mechanical properties of tissues and their ability to react to a deforming force. Depending on the nature of the deforming force, two elastography systems have been developed: strain elastography (SE) and shear wave elastography (SWE) [7]. SE is a qualitative or semi-quantitative technique that measures the deformation of the tissues by applying pressure with a probe on the body surface, and then displays the strain as an elastogram, with colors representing the degree of deformation; however, these evaluations are highly dependent on the degree and intensity of the compression [7] and are subject to a certain degree of inter-operator variability [8]. On the other hand, SWE uses a focused acoustic impulse to generate shear waves in the target tissue, the speed of which (SWS, measured in m/s) is directly proportional to tissue stiffness. Moreover, SWE techniques can be divided in focal quantitative techniques (point shear wave elastography, pSWE) and bidimensional quali-quantitative techniques (bidimensional shear wave elastography, 2D-SWE) [9,10]. Compared to SE, SWE techniques are more repeatable. However, all elastographic techniques require immobility of the patient; in fact, voluntary or involuntary motion (e.g., respiratory movements) may lead to artifacts and represents an issue for application in the veterinary field [11]. Different from other imaging techniques that assess morphology, ultrasound elastography is the only imaging technique capable of assessing tissue stiffness [7]. It increases the accuracy of ultrasound and enables the evaluation of malignant lesions, as malignancies are often stiffer than benign lesions and healthy tissues. These features allow clinicians to avoid unnecessary biopsies and to perform targeted ones [12,13].

Elastography has been widely used in human medicine to assess testicular stiffness both in normal conditions [14,15,16] and in various testicular pathologies, such as infertility [17,18,19,20], varicocele [21,22,23,24,25,26,27], testicular tumors [28,29,30], and testicular torsion [31,32]. Both SE and SWE techniques [33,34,35] have demonstrated clinical efficacy in distinguishing non-neoplastic focal testicular lesions from neoplasms and benign from malignant tumors [22,36]. In addition, increased testicular stiffness has been observed in association with abnormal sperm parameters, such as decreased sperm count [18,37].

In veterinary medicine, the use of SWE for the assessment of focal lesions in several organs has been reported. In canine mammary glands, benign neoplasms were softer than malignant ones [38,39,40], although SWE was not specific for the differentiation of carcinoma type or grads [41]. In dogs with head and neck cancer, higher SWS values were found in metastatic medial retropharyngeal and mandibular lymph nodes [42]. Moreover, 2D-SWE showed a diagnostic accuracy of 97% in the differentiation of malignant and benign splenic lesions in dogs, with malignant lesions being stiffer than benign ones and healthy parenchyma [43]. However, studies on the use of elastography for the assessment of testicular stiffness are still limited. In dogs, SE showed significant correlations between testicular stiffness and the quality of the semen collected from the epididymis [44], and SWE baseline values for healthy testes were reported both for pSWE and 2D-SWE [45,46]. In addition, some testicular abnormalities were described by pSWE [47], and 2D-SWE has been applied to differentiate leydigomas from non-neoplastic testicular lesions, showing significant differences [48]. However, the number of dogs and lesions evaluated is still limited and the diagnostic potential of SWE techniques in the assessment of testicular disease must be better addressed.

The aim of this study was to apply 2D-SWE and pSWE for the evaluation of testicular abnormalities in dogs, to describe their elastographic features using a combined approach of B-mode, power and color Doppler, 2D-SWE, and pSWE ultrasound techniques, supported by the histological diagnosis of the lesions.

## 2. Materials and Methods

### 2.1. Ethical Approval

All treatments, housing, and animal care followed European Regulation (EU) 2019/1010 [49]. The Ethics Committee of the Department of Veterinary Medicine and Animal Productions at the University of Messina, Italy (protocol n. 051/2021), approved the protocol and procedures. Informed consent was obtained from each dog owner before its inclusion in the study.

### 2.2. Animals Enrolled

This study, which took place between May 2022 and May 2023, involved client-owned dogs admitted in a private veterinary facility (Clinica Veterinaria Camagna-VetPartners, Reggio Calabria, Italy) for bilateral orchiectomy due to an evident testicular disease, diagnosed by clinical and/or ultrasound examination.

Inclusion criteria comprised the presence of at least one testicular ultrasound abnormality, with the exclusion of alterations affecting undescended testes (inguinal and/or abdominal cryptorchidism). In fact, we hypothesized that cryptorchid testes could receive external compression from adjacent structures, which may cause an artifactual increase in their stiffness. The enrollment occurred after written informed consent was obtained from the owners of each patient.

### 2.3. Ultrasound Procedures

Before surgical excision, the testes were examined by B-mode ultrasound, power and color Doppler ultrasound, and SWE (2D-SWE and pSWE) using a Mindray DC-80A ultrasound machine (Mindray Medical Italy S.R.L. Via Leonardo da Vinci, 158-20090 Trezzano sul Naviglio, Italy) and a 3–12 MHz linear probe. For examination, the dogs were positioned in lateral recumbency without any pharmacological restrictions and scrotal hair was shaved.

B-mode ultrasound was applied to evaluate the location, shape, size, margins (regular or irregular), echogenicity (hyperechoic, hypoechoic, isoechoic, anechoic, mixed, or complex), and echotexture (homogeneous, finely inhomogeneous, or inhomogeneous) of the testes and lesions. “Mixed” echogenicity was used to describe parenchymal structures with different echogenicity; “complex” echogenicity was used to describe tissue alterations characterized by solid and liquid components. Qualitative color and power Doppler ultrasound were used to detect the presence and distribution of blood flow of the lesions. In every Doppler examination, the pulse repetition frequency (PRF) and gain were set on 1.0 kHz and 50%, respectively.

For SWE evaluation, the scrotum was covered with a thick layer of ultrasound gel in order to obtain the best contact surface, and care was taken to apply the minimum pressure with the probe to avoid pre-compression artifacts [9,10]. As recommended by human guidelines, quantitative measurements were expressed as SWS in m/s [9].

For 2D-SWE, the FOV size was adjusted to include the entire testis within it, and images were acquired and stored when the M-STB index was at least 4 points. For qualitative image evaluation, the degree of elastic deformation of the testis was determined according to a color scale ranging from blue (elastic tissues; 0 m/s) to red (hard tissues; 5 m/s). For the quantitative assessment of focal lesions, two round ROIs were selected within the elastogram, one within the lesion area and one within an area showing a normal appearance (no evident ultrasound changes, NEUC), maintaining the same size and the same depth, when feasible [10]; for the assessment of diffuse lesions, a single central ROI was selected, including the largest possible area free from artifacts. To avoid artifacts, the ROIs were positioned far from vascular and/or cystic structures. The mean SWS and standard deviation (SD) within the ROIs were automatically calculated by the software. The assessment of the contralateral healthy testicle was performed, as a control, by positioning two pairs of ROIs above and below the mediastinum and obtaining the mean SWS.

For pSWE, 5 × 5 mm ROIs were selected and images were acquired with an M-STB index of at least 4/5 points and an IQR/Median ratio of ≤15% [9,10]. In the case of focal lesions, one ROI was placed at the lesion and another ROI was placed in an NEUC area of the testis, at the same depth, as a control, and at least five pairs of measurements were acquired. In the case of diffuse testicular alterations, the ROIs were placed in different portions of the testis and at least 5 measurements were acquired. For pSWE of healthy testes, the ROIs were placed above and below the mediastinal line and at least 5 acquisitions were obtained. All images were reviewed blinded to histological information.

### 2.4. Histopathological Examination

Following surgical excision, the testes were deposited in 10% buffered formalin, then cut, embedded in paraffin, and sectioned at 4 μm. Sections were stained with hematoxylin and eosin and examined for abnormalities of the testicular parenchyma. Final diagnosis of testicular lesions was made by histopathological evaluation, reviewed by a single veterinary pathologist blinded to the ultrasound imaging findings and clinical diagnosis.

After histological analysis, the data were grouped as: normal, NEUC, orchitis (with different subgroups according to the histological diagnosis), neoplasia (with different subgroups according to the histological diagnosis).

### 2.5. Statistical Analysis

The statistical analysis was performed using Jamovi software (Version 2.3.28.0 for MacOS). Quantitative variables are reported as mean ± standard deviation (SD), median, and range values, while categorical data are reported as frequency.

The normality of the distribution of quantitative variables was analyzed using the Shapiro–Wilk test, and positive or negative results were followed by parametric and non-parametric data analyses, respectively.

Comparisons of SWE parameters between different testicular lesions and with normal testes were performed using ANOVA (Kruskall–Wallis test), followed by the Dwass–Steel–Critchlow–Flinger post hoc test for pairwise comparisons. The significance threshold was *p* < 0.05.

## 3. Results

### 3.1. Animals

Eighteen male dogs of different breeds and aged between 4 and 14 years (mean 9.8 ± 2.4 years) met the inclusion criteria for this study (Table 1). Since five dogs (dog numbers 3, 4, 13, 16, and 18) were monolateral cryptorchid, 31 testes were included for the SWE examination.

### 3.2. Histopathological Diagnosis

Thirty-one testes underwent histopathological examination, which confirmed normality in 12 testes and diagnosed 14 neoplastic diseases (7 Leydig cell tumors, 3 diffuse seminomas, 2 intratubular seminomas, 1 intratubular and diffuse seminoma, and 1 poorly differentiated round cell tumor), and 5 orchites (2 interstitial lymphocytic, 2 purulent, and 1 pyogranulomatous orchiepididymitis).

### 3.3. B-Mode and Doppler US Examinations

Results of B-mode and Doppler US are shown in Table 2 and Table 3.

**Figure 1:**
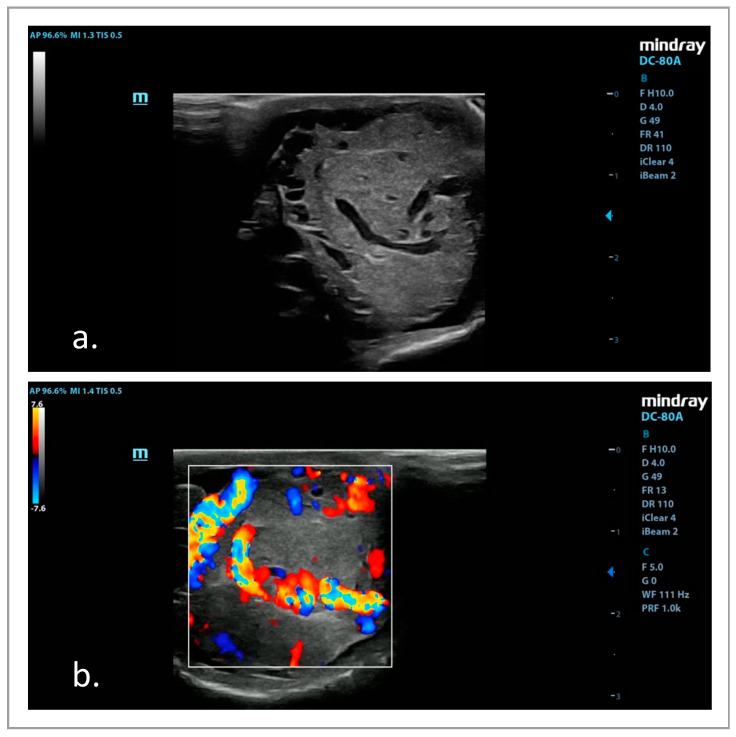
Ultrasound image of a canine testis affected by a Leydig cell tumor, with B–mode (**a**) and color Doppler ultrasound (**b**).

The average testicular size (length × height) was 29 ± 3 × 18 ± 3 mm in normal testes, 40 ± 9 × 28 ± 9 mm in testes with orchitis, 32 ± 9 × 21 ± 9 mm in testes with Leydig cell tumors, and 40 ± 11 × 29 ± 8 mm in testes with seminomas. The size of the testis affected by the round cell tumor was 57 × 37 mm. Normal testes were isoechoic with a finely inhomogeneous echotexture; testes with orchitis showed isoechoic (3/5) and mixed (2/5) echogenicities and finely inhomogeneous (3/5) and inhomogeneous (2/5) textures; seminomas had isoechoic (2/6), mixed (2/6), hypoechoic (1/6), and complex (1/6) echogenicities and inhomogeneous (4/6), homogeneous (1/6), and finely inhomogeneous (1/6) echotextures; leydigomas (Figure 1a) showed isoechoic (4/7), complex (2/7), and mixed echogenicities and homogeneous (3/6), inhomogeneous (3/6) and finely inhomogeneous (1/7) textures. The testis with the round cell tumor showed diffuse hyperechogenicity and a finely inhomogeneous echotexture.

Eleven focal lesions were present (Table 3). Among them, 3 were orchites, with an average size of 19 ± 8 mm, showing hypoechoic, iso-, and hyperechoic echogenicities and inhomogeneous, finely inhomogeneous, and homogeneous textures, respectively; 3 were seminomas, of 20 ± 25 mm in average size, respectively with hypoechoic echogenicity and a homogeneous texture (n. 2) and mixed-hyperechoic echogenicity and a finely inhomogeneous echotexture; 3 were Leydig cell tumors, with an average size of 16 ± 9 mm, showing hypoechoic, isoechoic, and complex echogenicities and homogeneous, finely inhomogeneous, and inhomogeneous textures, respectively; and the remaining one was the round cell tumor, measuring 14 mm in diameter, with a hyperechoic border, an isoechoic echogenicity, and finely inhomogeneous content.

Doppler ultrasound (Figure 1b) showed a peri- and intralesional vessel distribution in Leydig cell tumors, whereas it was absent or perilesional in focal orchites or regularly distributed in diffuse orchites. Seminomas exhibited an intralesional pattern, except one showed peri-and intralesional distribution. Normal testes showed broad vessel distribution. The round cell tumor showed an intralesional blood flow pattern.

### 3.4. SWE Examinations

#### 3.4.1. 2D-SWE

**Figure 2:**
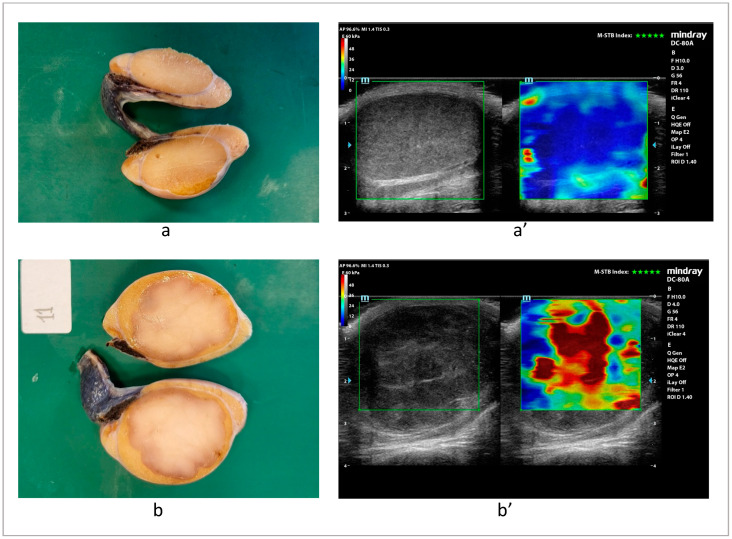
Gross anatomy (**a**,**b**) and 2D-SWE (**a’**,**b’**) appearance of a pair of testes, one being healthy (**a**,**a’**) and one affected by diffuse seminoma (**b**,**b’**).

Thirty-one testes were studied by 2D-SWE (Figure 2).

**Scheme 1:**
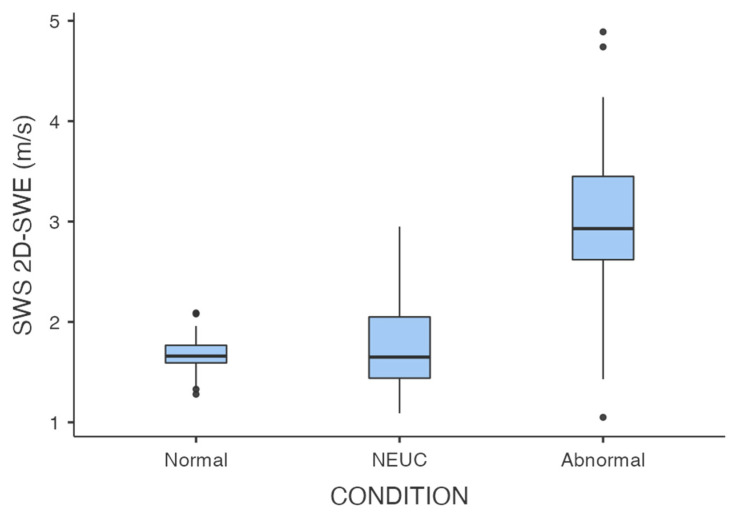
Box plot of SWS values measured by 2D-SWE in normal, NEUC, and abnormal testes. The boxes and vertical lines represent, respectively, the 95% confidence interval for the mean and the range values; horizontal lines represent median values; spots represent outlier values.

The mean SWS values obtained by 2D-SWE were significantly higher in diseased testes than in normal and NEUC ones (Table 4; Scheme 1). The mean SWS values obtained by 2D-SWE according to the histopathological diagnosis are shown in Table 5.

Significant differences were observed between the SWS values measured by 2D-SWE in normal testes, NEUC areas, orchites, leydigomas, and seminomas (*p* < 0.001). Pairwise post hoc tests revealed that the SWS values in normal testes were significantly different than the SWS values in leydigomas (*p* < 0.001), in diffuse seminomas (*p* = 0.002), in intratubular seminomas (*p* = 0.005), and in purulent orchites (*p* = 0.002). The SWS values in NEUC areas were significantly different from the SWS values in leydigomas (*p* < 0.001), diffuse seminomas (*p* = 0.003), and purulent orchites (*p* = 0.016). Furthermore, the SWS values did not differ significantly among orchites, and the SWS values in orchites did not differ significantly from the SWS values in leydigomas and seminomas, nor there were any significant differences between the testicular SWS values in seminomas and leydigomas.

#### 3.4.2. pSWE

**Figure 3:**
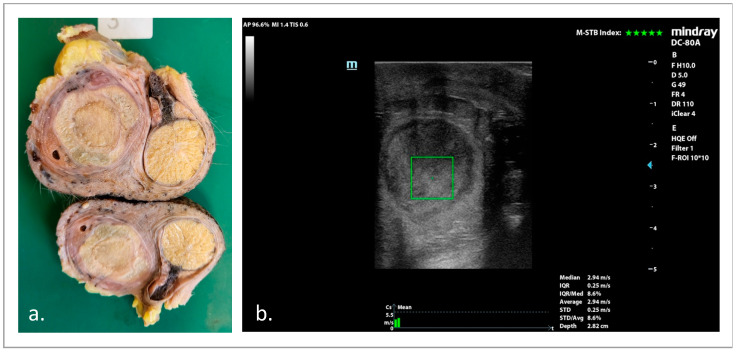
Appearance of gross anatomy (**a**) and ultrasonographic pSWE (**b**) in a dog with purulent orchitis. The green square represents the region of interest (ROI) in which the SWS is measured.

Due to incompliance by three dogs, twenty-eight testes were evaluated with pSWE (Figure 3).

**Scheme 2:**
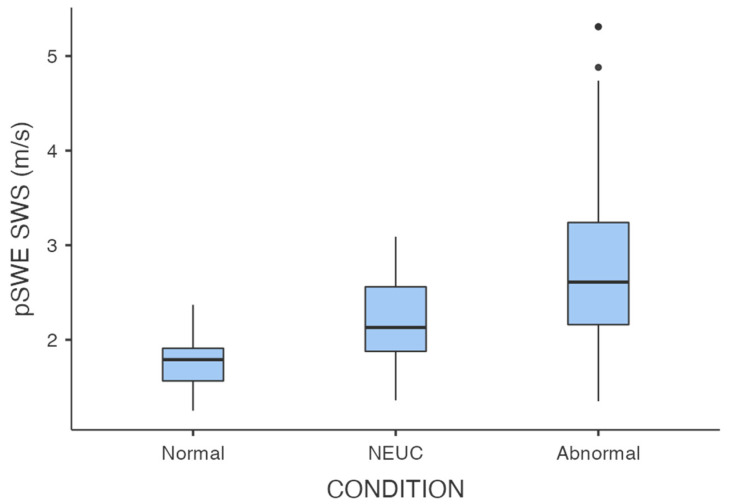
Box plot of average SWS values measured by pSWE in normal, NEUC, and abnormal testes. The boxes and vertical lines represent, respectively, the 95% confidence interval for the mean and the range values; horizontal lines represent median values; spots represent outlier values.

The mean SWS values obtained by pSWE were significantly higher in diseased testes than in NEUC areas and normal testes; NEUC areas showed significantly higher stiffness than normal testes (Table 6; Scheme 2). The mean SWS values obtained by pSWE are shown in Table 7.

Nonparametric one-way ANOVA (Kruskal–Wallis test) between normal, NEUC, and pathological testes revealed significant differences (*p* < 0.001), confirmed by the post hoc pairwise comparisons (*p* < 0.001).

Significant differences were found between the SWS values in normal testes, NEUC areas, orchites, and testicular tumors (*p* < 0.001); pairwise post hoc tests confirmed the statistical significance of these differences (*p* < 0.001), except for the SWS values in orchites and tumors, which did not differ significantly (*p* = 0.999).

Nonparametric ANOVA (Kruskal–Wallis test) showed significant differences among the SWS values in normal testes, NEUC areas, different orchites, and neoplastic lesions. In post hoc comparisons, the SWS values in normal testes were significantly different from the SWS values in leydigomas, seminomas (either intratubular, diffuse, or mixed), and purulent and pyogranulomatous orchites (*p* < 0.001), whereas no differences were found with the SWS values in interstitial lymphocytic orchites (*p* = 0.077). Significant differences were found in the SWS values in NEUC areas compared to those in leydigomas (*p* = 0.041), diffuse seminomas (*p* = 0.001), intratubular and diffuse seminoma (*p* < 0.001), and purulent and pyogranulomatous orchites (*p* < 0.001). The SWS values in leydigomas were significantly different from the SWS values in diffuse seminomas (*p* = 0.002), intratubular and diffuse seminoma (*p* < 0.001), and pyogranulomatous orchites (*p* < 0.001). The SWS values in intratubular seminomas were significantly different than the SWS values in intratubular and diffuse seminoma and pyogranulomatous orchites (*p* < 0.001). In both diffuse and intratubular and diffuse seminomas, the SWS values were significantly different than those in purulent (*p* = 0.016; *p* < 0.001, respectively) and pyogranulomatous (*p* = 0.039; *p*= 0.002, respectively) orchites.

## 4. Discussion

In this study, we enrolled 18 dogs affected by orchitis, seminomas, Leydig cell tumors, and a round cell tumor. Neoplastic processes represented the majority of our findings (56%), followed by normality (24%) and orchitis (20%). Among testicular tumors, Leydig cell tumors (n = 7) and seminomas (n = 6) were the most represented, and only one poorly differentiated round cell tumor was observed. No Sertoli cell tumors were found. Leydig cell tumors and seminomas are known to be the most common in scrotal testes, whereas Sertoli cell tumors are the most common in cryptorchid testes [50]. In our study, we did not detect any cases of Sertoli cell tumors likely due the small size of our sample, combined with the lower prevalence of this tumor compared to the others [50,51,52]. The mean age of occurrence of testicular tumors is 10 years [53], and the mean age of our population was consistent with these records.

Leydig cell tumors can vary in size from one to several centimeters; they are usually yellow-orange tumors, often round and encapsulated, soft and protruding when cut, containing serous or serosanguineous fluid. They can be solitary or multiple, uni- or bilateral [53,54]. They can present under three main histological patterns: the solid diffuse type, characterized by a cellular arrangement in sheets or chords, separated by thin septa; the pseudo-adenomatous type, with groups of cells surrounding hollow spaces containing acidophilic staining material; and the cystic vascular (or Angiomatoid) type, with few tumoral cells of various shape, organized into interconnecting strands surrounding spaces filled with pink-stained fluid and erythrocytes [54].

Seminomas are germ cell tumors commonly observed in old dogs. They are variable in size (1–10 cm) and can be solitary or multiple, and they often affect cryptorchid testes. Macroscopically, they can present as bulging and soft ivory-colored masses, variable in size, sometimes homogeneous or lobulated on the cut surface [53,54,55]. They can be classified into intratubular or diffuse histological types; they start with intratubular growth, which eventually invades the stroma and becomes the diffuse type [54]. They generally undergo a slow growth rate for a long time, then suddenly increase and may become large, necrotic, and/or hemorrhagic masses [56].

Inflammatory processes have a wide variety of presentations, from mild involvement, similar to degenerative processes, to suppurative and necrotic demolition; the testes can be increased in size during acute processes, or smaller and fibrotic in late chronic stages [56]. Lymphocytic orchitis is considered an immune-mediated disorder characterized by lymphocytic and plasmacytic infiltration of the testicular parenchyma, with immunoglobulin deposition in the seminiferous tubules. This leads to destruction of the seminiferous tubules, loss of Leydig cells, and development of antisperm antibodies. Consequently, the affected testes often become smaller and softer [55].

Degenerative processes can often affect testes since the seminiferous epithelium is susceptible to external and internal insults. Secondary tubular degeneration often occurs as a consequence of neoplastic invasion and/or inflammatory damage [55,56]. When chronic degeneration is a consequence of inflammation, evident processes of dystrophic calcification and fibrosis are observed; the testes become small, firm, lumpy, and inelastic. On the other hand, chronic degeneration caused by non-inflammatory insults and idiopathic degeneration induces a progressive loss of consistency and shrinkage of the testicles, leading to testicular atrophy; in the most severe forms, only the epididymides can be detected upon scrotal palpation [55].

Chronic testicular inflammation usually has a slow, low-grade, non-suppurative course that progresses to fibrosis, which causes loss of function and sperm production of the seminiferous tubules [55].

In our study, on B-mode ultrasound, the testes affected by seminomas were larger than normal ones and ones affected by Leydig cell tumors. This finding could be observed also when considering only focal lesions. The testis affected by the round cell tumor was the biggest observed in our population, showing a focal, encapsulated lesion, surrounded by a diffuse hyperechogenic, finely inhomogeneous alteration of the testicular parenchyma. This focal lesion was smaller than focal seminomas and leydigomas and represented an organized part of the overall tumor, which involved almost the entire testicular parenchyma. The echogenicity and echotexture of diffuse alterations were variably altered in both seminomas and leydigomas, while in focal lesions, the echogenicity was also variable, whereas the texture consistently ranged between finely inhomogeneous to homogeneous. These findings reflect how testicular tumors, although arising from different cellular lines, can have an overlapping ultrasonographic appearance [57].

Studies on the vascular perfusion of canine testicular lesions have been previously performed and agree on the controversial role of Doppler ultrasound in the differential diagnosis of testicular tumors; intra- and perilesional flow patterns were observed in leydigomas, seminomas, sertoliomas, and mixed cell tumors, without significant differences [57,58,59]. However, Bigliardi et al. (2019) [59] observed more pronounced peripheric and intralesional vascular signals in neoplasms compared to inflammatory and degenerative lesions [59], and Orlandi et al. (2022) [57] described Doppler ultrasound vascular patterns in canine testicular tumors, reporting that perilesional and perilesional/intralesional blood flow was more frequent in leydigomas and an intralesional blood flow pattern in seminomas [57]. Our results are consistent with these findings, since the leydigomas and seminomas found in our population showed peri- and intralesional vessel distribution. The round cell tumor showed an intralesional pattern; unfortunately, further immunohistochemical classification of the tumor was not feasible due to a sample preservation issue. Therefore, it was not possible to compare this result with previous bibliographic data.

2D-SWE was able to distinguish normal testes and diseased testes. However, it could not help in discriminating testes affected by orchitis from the ones with testicular neoplasias. Also, the testicular SWS values did not differ between the different kinds of testicular neoplasias observed. This may be due to the histological behaviors (e.g., fibrosis, necrosis, etc.) that can occur in all of these pathologic processes, which may influence stiffness in several ways. In fact, seminomas can show necrosis and hemorrhage [56], and leydigomas can be either compact or contain serosanguineous cysts [54]. All of these features can counterbalance cellular growth, influencing the overall SWS values in the lesions. As previously suggested by Gloria et al. (2023) [44], testicular stiffness (evaluated in their study as the elastographic index) may be the result of the balance between different testicular alterations; in their study, they suggested that an increased stiffness due to connective tissue deposition could be hidden by a loss of seminiferous tissue which, by contrast, would decrease testicular stiffness [44]. However, it must be considered that the authors did not evaluate testes affected by neoplastic or inflammatory diseases; thus, their histopathological findings are not comparable with ours. Our results are partially in contrast with those of Glińska-Suchocka et al. (2014) [48], who found significantly higher stiffness in six Leydig cell tumors (average 91.85 kPa = 5.53 m/s, range 52.3–131.4 kPa = 4.18–6.62 m/s) compared to three non-neoplastic testicular diseases (average 11.25 kPa = 1.94 m/s, range 6.1–16.4 kPa = 1.43–2.34 m/s) [48]. While our results on orchites (1.91 ± 0.96 m/s on five subjects) were consistent with theirs, the average SWS value measured in our study for leydigomas was much lower (2.86 ± 0.55 m/s), despite the similar sample size (7 dogs). However, the authors collected three quantitative measurements from each dog, without specifying the criteria for the sample acquisition. Therefore, there might be procedural differences that led to this inconsistency. Moreover, neither study performed further characterization of the histological type, which could have determined the overall stiffness. This limitation is particularly significant given the small sample sizes evaluated in both studies.

To the best of our knowledge, this is the first study reporting the elastographic evaluation of canine seminomas. In this regard, the difference that we observed between intratubular seminomas and the diffuse type may reflect the histological differences between these two forms of the same tumor: intratubular seminomas affect only the germinative epithelium and represent an early neoplastic stage, confined in seminiferous tubules. Probably, this stage affects testicular stiffness in a minor entity compared to the diffuse stage which, by contrast, is characterized by major invasion of the testicular stroma and surrounding structures [54].

As well as 2D-SWE, pSWE could help in distinguishing normal and diseased testicular tissues; however, it could not distinguish inflammatory from neoplastic disease. NEUC areas showed differences with normal, inflammatory, and neoplastic tissues, probably due to a compressive effect of the lesion on the surrounding structures, or perhaps due to the presence of false negatives in the NEUC group, as they looked like normal parenchyma by B-mode ultrasound. Moreover, using pSWE, the average SWS value in normal testes was slightly lower than our previous findings [46]. However, this could be due to the different sample sizes (36 healthy testes vs. 12 normal ones) and the different inclusion criteria adopted in these two studies. In fact, in the first study, our focus was on the fertility of the dogs (which was guaranteed by the semen examination), whereas in this study, the integrity of the testicle examined was guaranteed by the histopathological examination. In the first study, we may have also included testes with microscopic modifications that, although not altering the quality of the semen, potentially could have increased testicular rigidity (the only way to verify this would have been histology, but since we were working on fertile breeders, there were no clinical indications to perform biopsy); anyway, those SWS values, although slightly higher, were associated with fertility and testicular functional integrity. On the other hand, in this work, the physiological testes were included as controls for the pathological ones, and we did not aim to report a reference parameter. In fact, since we did not perform semen examinations, we cannot know whether, in addition to anatomical integrity, functional integrity was also maintained. In summary, the first study reports the standard of the healthy testicle from a functional point of view (more useful for clinical-andrological purposes), while the values obtained in this study reflect the healthy testicles from a purely anatomical/structural point of view.

As well as with 2D-SWE, the first pSWE values for seminomas are reported in our study. Previous reports of SWS values of testicular changes in dogs by pSWE were testicular degeneration (0.97 ± 0.08 m/s), testicular atrophy (2.00 ± 0.35 m/s), testicular hypoplasia (0.82 ± 0.2 m/s), testicular cysts (1.32 ± 0.18 m/s), orchitis (2.68 ± 0.42 m/s), interstitial cell tumors (3.32 ± 0.65 m/s), sertoliomas (2.99 ± 0.07 m/s), and leydigomas (2.73 ± 0.37) [47]. An important difference in the methods is that the authors included cryptorchid testes, while we excluded those cases in order to avoid any bias related to the different location and/or compressing factors exerted by the surrounding structures. Moreover, their sample, although similar to ours (36 testes), was differently represented; the authors evaluated only 3 orchites and 4 tumors (2 “interstitial cell tumors”, 1 sertolioma, and 1 “leydigoma”). They only performed descriptive statistics and did not perform statistical comparisons of their results. Nonetheless, their results for orchitis were similar to ours (2.77 ± 0.91 m/s) [47]. It is not clear which is the actual SWS value for the leydigoma (or interstitial cell tumor) since they reported the tumor twice with different SWS values.

Diagnostic accuracy of elastography in the evaluation of testicular lesions is also challenging in human medicine [60]. Dikici et al. (2016) [29] could differentiate germ cell tumors according to their stiffness, since seminomas were softer than non-seminomatous germ cell tumors (e.g., teratomas), which are characterized by the presence of bony or cartilage parts [29]. Under progressive torsion, testes showed an initial increase in stiffness until testicular necrosis occurred, then the testes became softer [61].

In a review of multiparametric ultrasound of human scrotal diseases, Bertolotto et al. (2018) [62] reported inconsistency in the literature, with some studies stating that about 100% of malignant lesions appeared stiff by elastography, and other investigations showing soft malignant lesions and stiff non-neoplastic lesions (e.g., cysts, hematoma, infarction, rete testis, and scars) [62]. The assumption that malignant tumors are stiffer than benign alterations and normal parenchyma can have some exceptions, like lesions with a high vessel density showing similar stiffness as the surrounding tissue and malignant lesions showing both necrotic and fibrotic processes [60]. Some scholars consider that elastography alone is not sufficiently accurate in the discrimination of scrotal lesions but acknowledge its diagnostic potential when performed as a part of a multiparametric ultrasound [33,62].

There were no significant differences between the SWS values in normal and NEUC testes by 2D-SWE, whereas they were present when performing pSWE. The reason for this result can be that when placing the ROI in a 2D-SWE picture, the operator is influenced by both the B-mode image and the elastogram, while the only B-mode appearance can be used as a guide when choosing ROIs in pSWE mode. Therefore, when selecting the ROIs in 2D-SWE, the operator chose “NEUC” areas with both a normal appearance in B-mode and a “blue” color in the elastogram; conversely, during the pSWE examination, the operator could rely only on the normal B-mode ultrasound appearance for ROI placement. Thus, the operator could have selected false negative areas in the pSWE exams. Furthermore, perilesional areas, even if histologically intact, may result from a compressive effect by the adjacent lesion. This could have determined an increased SWS value in the NEUC areas. Furthermore, in pSWE examinations, the ROIs are placed in real-time assessment, while the selection of ROIs in 2D-SWE is performed in post-processing; however, this may have influenced the results only partially since the operator was unaware of the final diagnosis of the lesions during both the examinations and the subsequent review of the images. The different SWS values obtained by pSWE between normal and NEUC areas may reflect the lower sensitivity of the only B-mode assessment compared to SWE, which is able to detect increased stiffness before the appearance of ultrasonographic changes. However, measurements in perilesional areas could have a poor clinical role and may represent only a comparison with abnormal areas.

This research has some limitations. First, the small number of testicular alterations observed, in some cases only one (e.g., the round cell tumor). Moreover, also in this study, incompliance by the patients did not allow the operator to perform both 2D-SWE and pSWE examinations in all of the dogs enrolled. A bigger and diverse sample is recommended in order to broaden the knowledge of SWE evaluation of testicular lesions in dogs. Another limitation was the lack of quantitative Doppler evaluations (such as the pulsatility index of the testicular artery), which could have allowed for a more detailed characterization of the vascularization of lesions. Further studies should evaluate quantitative flow assessment and SWE parameters. However, to date, the number of canine testicular tumors evaluated by SWE is very low. Our work has not only expanded this number but has added new evidence for seminomas and other testicular abnormalities.

## 5. Conclusions

In conclusion, by revealing a neat difference in SWS values between healthy and damaged testicular tissues, SWE techniques can aid clinicians in the assessment of testicular alterations, providing a supporting tool in the diagnostic process. However, testicular diseases present under various histological types, with a wide range of overlapping and nonspecific stiffness values. Therefore, the clinical utility of SWE techniques for the differential diagnosis of testicular alterations demonstrates potential, and the relationship between specific histological types and their corresponding SWE values deserves further investigation.

## Figures and Tables

**Table 1:** Breed and age of dogs enrolled in the study.

Dog	Breed	Age (years)
1	Mixed	8
2	Mixed	11
3 ^(c)^	Airedale Terrier	11
4 ^(c)^	Mixed	8
5	Mixed	9
6	Shih Tsu	11
7	German Shepherd	10
8	Mixed	10
9	Fox Terrier	14
10	Mixed	14
11	French Bulldog	7
12	Mixed	9
13 ^(c)^	Mixed	11
14	German Wirehaired Pointer	11
15	Mixed	12
16 ^(c)^	Mixed	9
17	American Staffordshire	4
18 ^(c)^	Pointer	8

^(c)^ = monolateral cryptorchid dog: only the scrotal testis was evaluated in this subject.

**Table 2:** Results of the B-mode examination (size, shape, echogenicity, and echotexture), Doppler examination (flow pattern), histopathological examination of each of the examined testes, and 2D-SWE and pSWE elastography results (mean SWS).

		B-mode US				Doppler US	Histopathology	SWS (m/s)	
Dog	Testis	Size	Shape	Echogenicity	Texture	Pattern	Diagnosis	2D-SWE	pSWE
**1**	R	44 × 28	OS	Iso	FIE	Normal	Interstitial lymphocytic orchitis	3.73	4.75
	L	27 × 15	OS	Iso	FIE	Normal	Interstitial lymphocytic orchitis	1.59	1.83
**2**	R	38 × 27	RS	Hypo	IE	Intralesional	Intratubular and diffuse Seminoma	3.46	3.6
	L	25 × 15	OS	Iso	H	Normal	Normal	1.85	1.9
**3**	R ^(c)^	-	-	-	-	-	N.E.	-	-
	L	45 × 21	OS	ME	H	Normal	Normal	1.32	1.8
**4**	R	45 × 35	RS	CE	IE	Intralesional	Diffuse seminoma	3.04	N.E.
	L ^(c)^	-	-	-	-	-	N.E.	-	-
**5**	R	33 × 24	OS	Iso	HE	Intralesional	Intratubular seminoma	2.17	N.E.
	L	47 × 39	RS	Iso	IE	Absent	Purulent orchitis	2.7	3.03
**6**	R	27 × 15	OS	Iso	HE	Normal	Normal	1.92	1.84
	L	57 × 37	OS	ME/Hyper	IE	Intralesional	Poorly differentiated round cell tumor	2.98	N.E.
**7**	R	25 × 16	OS	Iso	HE	Normal	Normal	1.67	1.81
	L	58 × 40	IS	ME	IE	Intralesional	Diffuse seminoma	4.18	1.78
**8**	R	34 × 24	RS	ME	FIE	Perilesional	Pyogranulomatous orchiepididymitis	4.18	3.02
	L	28 × 16	OS	Iso	FIE	Normal	Normal	1.71	1.52
**9**	R	24 × 15	OS	Iso	HE	Normal	Normal	1.66	1.79
	L	23 × 12	OS	ME	IE	Peri-/intralesional	Leydigoma	2.63	2.54
**10**	R	27 × 16	OS	Iso	HE	Normal	Normal	1.61	1.76
	L	38 × 26	RS	CE	IE	Peri-/intralesional	Leydigoma	3.08	3.04
**11**	R	29 × 17	OS	Iso	HE	Normal	Normal	1.46	1.86
	L	40 × 29	RS	ME	IE	Intralesional	Diffuse seminoma	4.1	4.17
**12**	R	47 × 37	IS	CE	IE	Peri-/intralesional	Leydigoma	2.9	2.75
	L	26 × 16	OS	Iso	HE	Normal	Normal	1.68	1.82
**13**	R ^(c)^	-	-	-	-	-	N.E.	-	-
	L	23 × 13	OS	ME	HE	Peri-/intralesional	Leydigoma	3.08	2.32
**14**	R	25 × 15	OS	Iso	HE	Normal	Normal	1.62	1.77
	L	27 × 16	OS	Iso	FIE	Intralesional	Intratubular seminoma	2.77	2.32
**15**	R	26 × 16	OS	Iso	HE	Peri-/intralesional	Leydigoma	3.16	2.22
	L	30 × 25	OS	Iso	HE	Peri-/intralesional	Leydigoma	2.12	1.97
**16**	R	31 × 21	OS	Iso	HE	Normal	Normal	1.6	1.84
	L ^(c)^	-	-	-	-	-	N.E.	-	-
**17**	R	48 × 34	RS	ME	IE	Absent	Purulent orchitis	2.8	2.52
	L	30 × 20	OS	Iso	HE	Normal	Normal	1.6	1.74
**18**	R ^(c)^	-	-	-	-	-	N.E.	-	-
	L	36 × 21	OS	Iso	FIE	Peri-/intralesional	Leydigoma	2.77	3.03

^(c)^ = cryptorchid testis; R = right testicle; L = left testicle; OS = oval shape; RS = rounded shape; IS = irregular shape; Hyper = hyperechoic; Hypo = hypoechoic; Iso = isoechoic; ME = mixed echogenicity; CE = complex echogenicity; HE = homogeneous echotexture; IE = inhomogeneous echotexture; FIE = finely inhomogeneous echotexture; N.E. = not evaluable.

**Table 3:** B-mode (Number, size in mm, shape, margins, echogenicity, texture, and location) and Doppler (perfusion pattern) ultrasound features of focal lesions observed in the dogs enrolled, with the relative histopathological diagnosis and 2D-SWE and pSWE results.

		US of Focal Lesions							Doppler US	Histopathology	SWS (m/s)	
Dog	Testis	Number	Size (mm)	Shape	Margins	Echogenicity	Texture	Location	Pattern	Diagnosis	2d-swe	Pswe
1	L	1	12 × 11	IS	D	Hypo	IE	Central	Normal	Interstitial lymphocytic orchitis	3.73 (FL); 1.79 (NEUC)	4.75 (FL); 2.84 (NEUC)
5	R	1	3.92 ⊘	RS	D	Hypo	HE	Central	Intralesional	Intratubular seminoma	2.17 (FL); 1.4 (NEUC)	N.E.
5	L	1	28 ⊘	RS	D	Iso	FIE	Central	Absent	Purulent orchitis	2.7 (FL)	3.03 (FL)
6	L	1	14 ⊘	RS	D	Iso	FIE	Cranial	Intralesional	Poorly differentiated round cell tumor	2.98 (FL)	N.E.
7	L	1	58 × 40	IS	D	ME/Hyper	FIE	Periphery	Intralesional	Diffuse seminoma	4.18 (FL); 2.27 (NEUC)	1.78 (FL)
8	R	1	21 × 12	RS	PD	Hyper	HE	Central	Perilesional	Pyogranulomatous orchiepididymitis	4.18 (FL); 1.41 (NEUC)	3.02 (FL)
13	L	1	6 × 4	OS	D	Hypo	HE	Central-Caudal	Peri-/intralesional	Leydigoma	3.08 (FL)	2.32 (FL); 2.24 (NEUC)
14	L	1	8 × 7	IS	D	Hypo	HE	Central	Intralesional	Intratubular seminoma	2.77 (FL); 1.64 (NEUC)	2.43 (FL); 1.91 (NEUC)
15	R	1	9 ⊘	RS	PD	Iso	HE	Central	Intralesional	Leydigoma	3.16 (FL); 2.16 (NEUC)	2.22 (FL); 1.84 (NEUC)
15	L	1	24 ⊘	RS	D	Iso	FIE	Caudal	Peri-/intralesional	Leydigoma	2.12 (FL); 1.39 (NEUC)	1.97 (FL); 1.92 (NEUC)
18	L	1	18 ⊘	R	D	CE	IE	Central	Peri-/intralesional	Leydigoma	2.77 (FL); 1.64 (NEUC)	3.03 (FL); 2.61 (NEUC)

R = right testicle; L = left testicle; ⊘ = diameter; IS = irregular shape; RS = rounded shape; OS = oval shape; D = defined margins; PD = poorly defined margins; Hyper = hyperechoic; Hypo = hypoechoic; Iso = isoechoic; ME = mixed echogenicity; CE = complex echogenicity; IE = inhomogeneous echotexture; HE = homogeneous echotexture; FIE = finely inhomogeneous echotexture; FL = focal lesion; NEUC = area with no evident ultrasound change; N.E. = not evaluable.

**Table 4:** Mean ± standard deviation (SD), 95% confidence interval (CI) for the mean, median, and range SWS measured by 2D-SWE in normal, NEUC (no evident ultrasonographic changes), and abnormal testes.

2D-SWE	Condition	MEAN ± SD	95% CI	Median	Range
Mean SWS (m/s)	Normal	1.66 ± 0.20	1.59–1.73	1.66	1.28–2.09
	NEUC	1.78 ± 0.48	1.59–1.98	1.55	1.09–2.95
	Abnormal *	2.94 ± 0.8	2.75–3.14	2.93	1.05–4.89

* = statistically significant difference with SWS of Normal and NEUC (*p* < 0.001).

**Table 5:** Testicular SWS (mean ± SD, 95% CI for the mean, median, and range) values measured by 2D-SWE in canine testes according to the histopathological diagnosis.

DIAGNOSIS	Mean ± SD	95% CI	Median	Range
**Normal**	1.66 ± 0.20	1.59–1.73	1.66	1.28–2.09
**NEUC**	1.78 ± 0.48	1.59–1.98	1.55	1.09–2.95
**ORCHITES**	1.91 ± 0.96	2.49–3.34	2.91	1.05–4.24
**Interstitial lymphocytic orchitis**	2.87 ± 1.28	1.29–4.46	2.93	1.52–4.22
**Purulent orchitis**	2.75 ± 0.81	2.31–3.20	2.76	1.05–3.95
**Pyogranulomatous orchiepididymitis**	4.18 ± 0.09	13.35–5.00	4.18	4.11–4.24
**TUMORS**	2.96 ± 0.72	2.74–3.17	2.97	1.63–4.89
**Leydig cell tumor**	2.86 ± 0.55	2.63–3.09	2.97	1.63–3.59
**Diffuse seminoma**	3.83 ± 0.84	3.06–4.6	4.1	2.92–4.89
**Intratubular seminoma**	2.47 ± 0.61	2.04–2.91	2.66	1.64–3.54
**Intratubular and diffuse seminoma**	3.46 ± 0.01	3.33–3.59	3.46	3.45–3.47
**Round cell tumor**	2.98 ± 0.17	1.46–4.5	2.98	2.86–3.1

NEUC = no evident ultrasound changes.

**Table 6:** Mean ± standard deviation (SD), 95% confidence interval (CI) for the mean, median, and range SWS measured by pSWE in normal, NEUC (no evident ultrasonographic changes), and abnormal testes.

pSWE	CONDITION	MEAN ± SD	95% CI	MEDIAN	RANGE
Mean SWS (m/s)	Normal	1.76 ± 0.27	1.70–1.83	1.79	1.25–2.37
	NEUC	2.22 ± 0.43	2.12–2.33	2.13	1.36–3.09
	Abnormal	2.73 ± 0.80	2.63–2.83	2.61	1.35–5.31

**Table 7:** Testicular SWS values (mean ± SD, 95% CI for the mean, median, and range) measured by pSWE in canine testes according to the histopathological diagnosis.

*DIAGNOSIS*	*Mean ± SD*	*95% CI*	*Median*	*Range*
*Normal*	1.76 ± 0.27	1.70–1.83	1.79	1.25–2.37
*NEUC*	2.22 ± 0.43	2.12–2.33	2.13	1.36–3.09
***ORCHITES***	2.77 ± 0.91	2.58–2.96	2.69	1.45–5.31
*Interstitial lymphocytic orchitis*	2.61 ± 1.34	2.14–3.07	1.96	1.45–5.31
*Pyogranulomatous orchiepididymitis*	3.02 ± 0.43	2.84–3.20	3.09	2.07–3.92
*Purulent orchitis*	2.75 ± 0.45	2.58–2.92	2.82	2.07–3.67
***TUMORS***	2.71 ± 0.73	2.59–2.82	2.49	1.35–4.68
*Leydig cell tumor*	2.50 ± 0.57	2.39–2.61	2.38	1.35–4.68
*Intratubular seminoma*	2.43 ± 0.15	2.33–2.52	2.47	2.25–2.61
*Diffuse seminoma*	3.80 ± 0.95	3.23–4.38	4.16	1.71–4.48
*Intratubular and diffuse seminoma*	3.6 ± 0.12	3.52–3.67	3.62	3.32–3.73

NEUC = no evident ultrasound changes.

## Data Availability

The data presented in this study are available on request from the corresponding author due to privacy restrictions.
